# Four-Year Outcomes of Immediately Loaded Full-ArchMaxillary Dental Implants with Hybrid Versus Roughened Surfaces: A Split-Mouth Randomized Controlled Trial

**DOI:** 10.3390/jcm14227996

**Published:** 2025-11-11

**Authors:** David Offord, Nicola Kingsford, Maarten Glibert, Jeremy Pitman, Véronique Christiaens

**Affiliations:** 1 Private Practice, Vermilion the Smile Experts, 24 St John’s Road, Edinburgh EH12 6NZ, UK; 2 Department of Periodontology and Oral Implantology, Faculty of Medicine and Health Sciences, Oral Health Sciences, Ghent University, C. Heymanslaan 10, B-9000 Ghent, Belgium

**Keywords:** dental implants, implant supported denture, dental etching, alveolar bone loss, osseointegration, randomized controlled trial

## Abstract

**Background/Objectives:** The aim of this randomized control trial was to compare crestal bone levels from two implant types featuring surfaces of differing roughness after 4 years of follow up. The first implant type featured a hybrid surface: a minimally rough machined surface over the initial 3 mm between the prosthetic platform and the apex and a moderately rough grit-blasted surface over the remaining implant length. The second type featured a moderately rough grit-blasted surface from the implant collar to the apex and was considered as the control. **Methods:** Two of each implant type were placed in a split mouth sequence in the maxilla of 34 partially or fully edentulous patients in the original cohort. All implants were immediately loaded with either a fixed or removable denture. **Results:** Twenty-four patients with 97 implants were present at the 4-year recall, and 3 failed implants were recorded (implant survival rate = 96.9%). No statistically significant difference in crestal bone loss was found between the test and control implant types at the 4-year evaluation. **Conclusions:** Considering the sample size-related limitations of this randomized controlled trial, both hybrid and moderately rough implants showed minimal bone loss over a 4-year follow up period. While these results would suggest comparable stability over the longer term, the limited sample size prevents definitive conclusions from being made regarding clinical superiority and broader generalizability of the results. A larger, adequately powered study is warranted to confirm these findings.

## 1. Introduction

Full arch rehabilitation with an implant supported removable prosthesis, is common nowadays and well described in the literature. Implant survival in maxillary overdentures is predictable when using four or more implants, although most long-term follow-up studies only describe a delayed prosthetic loading protocol [1,2]. Studies reporting on immediate loading using implant supported overdentures, on the other hand, are less well described. There is evidence suggesting a predictable outcome in the mandible [2,3]. However, bone quality in the maxilla is known to be inferior to the mandible, thereby jeopardizing primary implant stability and potentially making an immediate loading protocol less predictable [4,5]. Unfortunately, there is limited published evidence on immediate loading of implant supported maxillary overdentures [6,7].

An evolution in implant design is the use of a modified implant surface. Implant surface roughness has been shown to enhance osseointegration and gives a more predictable treatment outcome in difficult conditions such as immediate loading and placement [8,9,10]. Implant surface roughness may be categorized as minimally, moderately rough, or rough depending on their arithmetical mean surface height, or *S_a_* values. Minimally rough surfaces have *S_a_* values between 0.5 µm and 1 µm; moderately rough surfaces have *S_a_* values between 1 µm and 2 µm; and rough surfaces have *S_a_* values greater than 2 µm [9]. A higher initial implant failure rate was reported in a systematic review from De Bruyn et al., when using minimally rough implants compared to moderately rough implants after immediate loading [6]. The initial failure rate of the minimally rough implants was even higher when placing the implants immediately into an extraction socket, whereas no difference in implant failure could be found when using moderately rough implants for these more critical loading and placing protocols [6]. Nagay & colleagues found no differences in implant survival when using anodized implants, regardless of the loading protocol (immediate vs. delayed) or prosthetic rehabilitation (fixed full prosthesis vs. overdenture) [11]. However, immediately loaded implants supporting a fixed prosthesis were found to have less crestal bone loss compared to implants with a delayed loading protocol. The opposite result was reported when considering implant supported overdentures, as immediate loading showed higher crestal bone loss than delayed loading [11]. These contradictory results, emphasize that the effect on implant treatment outcome of loading protocol and prosthetic rehabilitation is not yet fully clarified.

Another factor possibly affecting the treatment outcome is the use of pre-angulated implants. Sometimes a mismatch between the optimal surgical and restorative positions for an implant may arise, due to the natural angulation of the alveolar ridge or an important anatomical structure (for example, the maxillary sinus). Often, this mismatch may be corrected with an angulated abutment, but these abutments are often associated with an extra cost and are more prone to crestal bone loss [12]. An alternative solution to correct this mismatch is to use an implant with a built-in angulation correction (Co-axis^®^, Southern Implants Ltd., Irene, South Africa) [13]. A recent systematic review stated that this type of implant showed similar success rates with conventional implants in immediate loading and placement [14].

As mentioned, rougher implant surfaces have been proven to give a more predictable osseointegration [6,8,9,10]. On the other hand, rougher implant surfaces may enhance plaque and biofilm accumulation [15,16]. The latter may also result in a higher risk of infection of the gingival tissues surrounding the implant. In their systematic review, Doornewaard et al. also found greater crestal bone loss around rough and moderately rough implants after 5 years, compared to minimally rough or machined surface implants [9]. Hybrid surface implants feature a roughened implant body and a smoother section at the coronal aspect. These implants were designed to reduce the risks of peri-implant mucositis and peri-implantitis and to minimize late crestal bone loss without jeopardizing the osseointegration [9,10,17,18]. Multiple clinical studies comparing hybrid and fully moderately rough implants, found comparable implant survival in the long and the short term [19,20,21]. To our knowledge, there are no studies comparing clinical outcomes of hybrid surface implants with moderately rough surface implants in full arch immediate loading cases.

The primary aim of this randomized controlled trial was to compare crestal bone loss around immediately loaded hybrid and roughened implants supporting a full arch removable prosthesis. Plaque, bleeding on probing, and pocket depth served as secondary outcomes. The hypothesis of this randomized controlled trial is that hybrid implants provide comparable performance to moderately rough implants in terms of crestal bone loss.

## 2. Materials and Methods

### 2.1. Study Design and Patient Selection

The present study was conducted as a split-mouth, single blinded randomized controlled trial and reported according to the CONSORT guidelines (Table S2) [22]. The inclusion criteria were as follows:

- Partially and fully edentulous patients older than 21 years requiring full arch rehabilitation of the maxilla;
- Sufficient bone volume to place implants at least 4 mm in diameter and 10 mm long, respectively;
- Willingness to participate and complete the study and provide consent.

The exclusion criteria were as follows:

- Poor oral health;
- Active periodontal disease;
- Previous irradiation treatment to the head and neck;
- Immunosuppressed or immunocompromised;
- Bisphosphonate medication;
- Smoking > 10 cigarettes per day;
- Uncontrolled diabetes.

Randomization was performed using sequentially numbered, opaque, and sealed envelopes, and allocation of implants was concealed until the day of surgery. Sample size calculation was performed using SAS Power and Sample Size Calculator (version 9.4, SAS Institute, Cary, NC, USA) using an effect size of 1 mm and a standard deviation of 0.6 mm. The level of significance (alpha) was set at 0.05, and the statistical power (beta) was set at 0.8. Based on the parameters from Vervaeke et al. [23], a minimum sample size of 30 patients (and 120 implants) was required. To compensate for potential dropouts, the total number of patients to include was raised to 34. All patients were informed in detail about the treatment received and signed an informed consent form. The study was conducted in accordance with the principles described in the 1975 Declaration of Helsinki on clinical research in human subjects (as revised in 2008) [24] and was approved by the Southeast Scotland Research Service Area 2 (Reference No. 17/SS/0063) prior to commencement. The trial was also registered on the Standard Randomized Control Trial public database (ISRCTN57543596; trial approval date: 12 June 2017).

### 2.2. Surgical Protocol

**Figure 1:**
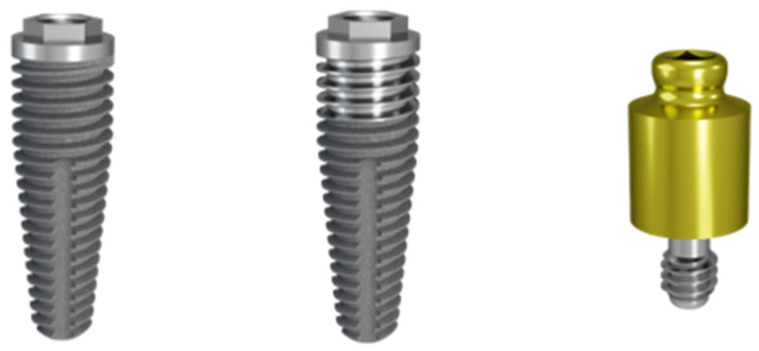
Examples of a Ø4 × 10 mm moderately rough implant (**left**) and a Ø4 × 10 mm hybrid implant with a 3 mm long minimally rough section at the coronal collar (**center**). Throughout the study, implants of both types with diameters of Ø4 mm, Ø5 mm, or Ø7 mm and lengths varying from 10 to 15 mm were used for both immediate and delayed implant placement. Each patient received an equal number of hybrid and moderately rough implants. Immediately after implant placement, a locator abutment (**right**) was placed on all implants. Images used with permission from Southern Implants Ltd.

All surgeries were performed by the same oral surgeon (DO), and prosthetic rehabilitation was performed by 1 of the 3 restorative dentists in the same practice. On the day of surgery, a sealed envelope containing instructions for which implant type to place in the anterior/posterior region of the first or second quadrant was opened. Four implants were placed in a split-mouth sequence in the maxilla of each patient: 2 control implants (=moderately rough surface) and 2 test implants (=hybrid minimally rough/moderately rough surface; MSC, Southern Implants Ltd., Pretoria, South Africa). The control implants had an alumina-blasted surface over the full length (*S_a_* = 1.3 µm; *S_dr_* = 60%) [25]. The test implants featured a machined surface over the initial 3 mm from the platform towards the apex (*S_a_* = 0.9 µm; *S_dr_* = 34%) [15], and an alumina-blasted surface for the remaining implant length (*S_a_* = 1.3 µm; *S_dr_* = 60%) [25]. All implants had an external hex prosthetic connection, with a diameter of 4, 5, or 7 mm and a length varying from 10 to 15 mm. If required for prosthetic reasons, implants with a pre-angled platform of 12° or 24° were placed (Co-axis^®^, Southern Implants Ltd., Pretoria, South Africa). The implants were inserted into either healed ridge or extraction socket sites according to the manufacturer’s instructions (Southern Implants Ltd., Pretoria, South Africa). Primary stability was evaluated by use of a manual torque wrench (Figure 1).

Self-aligning locator abutments were then immediately attached to the implants to load the interim prosthesis. Locator abutment height was based on the clinical presentation of each case, with careful consideration of the patient’s soft tissue phenotype. To ensure that the biological width around the implants was respected in all cases [23]. Since impressions were taken the day before, a temporary restoration was available at the moment of surgery. The temporary acrylic prosthesis was then placed, and the locator/housings picked up using UFI-GEL. Patients were instructed to retain the temporary prosthesis until the 2-week post-surgical follow up appointment. Postoperative home care and instructions for a soft diet were provided, and wound healing was assessed during the follow-up appointment. Patients could choose between a fixed (*n* = 10) versus a removable prosthesis (*n* = 21). Construction and delivery of definitive removable or fixed prostheses occurred between 5 and 17 months post-surgery. Patients were recalled for follow up as needed over the first 6 months after definitive rehabilitation.

### 2.3. Radiographic Analysis

Digital periapical radiographs were taken for each implant using standard settings (1–4 exposures of 60 kV, 2.5 mA for 0.13 s) with a handheld X-ray system (Nomad Pro, DEXIS, Quakertown, PA, USA) at implant placement (T_0_); 6–18 months after surgery (T_1_); and at 47–59 months (T_2_) after implant placement. The parallel long-cone technique combined with a guiding system (Schick, New York, NY, USA) was used to standardize the X-ray direction to be perpendicular to the sensor and parallel to the implant’s long axis. If the implant threads were not clearly visible, additional radiographs were obtained until the bone level could be assessed. Based on these radiographs, bone loss was measured using Image J software (version 1.53k, National Institutes of Health, Bethesda, MD, USA) [26] by two independent and blinded researchers (JP & MG). Each radiograph was calibrated using the known distance between two implant threads. Distance from the implant-abutment interface to the crestal bone was measured on the mesial and distal aspects at baseline and at T_1_ and T_2_.

### 2.4. Clinical Evaluation

Due to the COVID-19 pandemic, clinical evaluations were performed within the first 18 months after implant placement at T_1_ (6–18 months) and again at T_2_ (47–59 months). PPD (in mm), BOP (0 = no bleeding; 1 = bleeding), and PI (0 = no plaque; 1 = presence of visible plaque) were measured at 6 sites for each implant: mesial-buccal, buccal, distal-buccal, mesial-lingual, palatal, and distal-palatal. An average value per implant was calculated for each of these outcomes.

### 2.5. Statistical Analysis

Statistical analyses were conducted using SPSS version 29.0 (IBM SPSS Statistics for Windows, IBM Corp: White Plains, NY, USA.). Agreement between the 2 blinded examiners was evaluated using the intraclass correlation (IC) coefficient, and the average of the mesial and distal bone loss measurements was considered as the crestal bone loss level at baseline and follow-up. A linear mixed effects model was used to test for significant differences in crestal bone loss between the 2 implant types, taking possible interactions between the different variables into account. Implant type (hybrid vs. moderately rough), implant surgery (immediate vs. healed), graft (yes/no), built-in prosthetic angulation correction (yes/no), and final prosthetic rehabilitation (fixed vs. removable) were considered as fixed factors in the statistical model. The model was validated for linearity and homoscedasticity. Differences in terms of PPD, BOP, and PI between the implant types were assessed by using Mann–Whitney U tests. Statistical significance was set at *p* < 0.05.

## 3. Results

**Figure 2:**
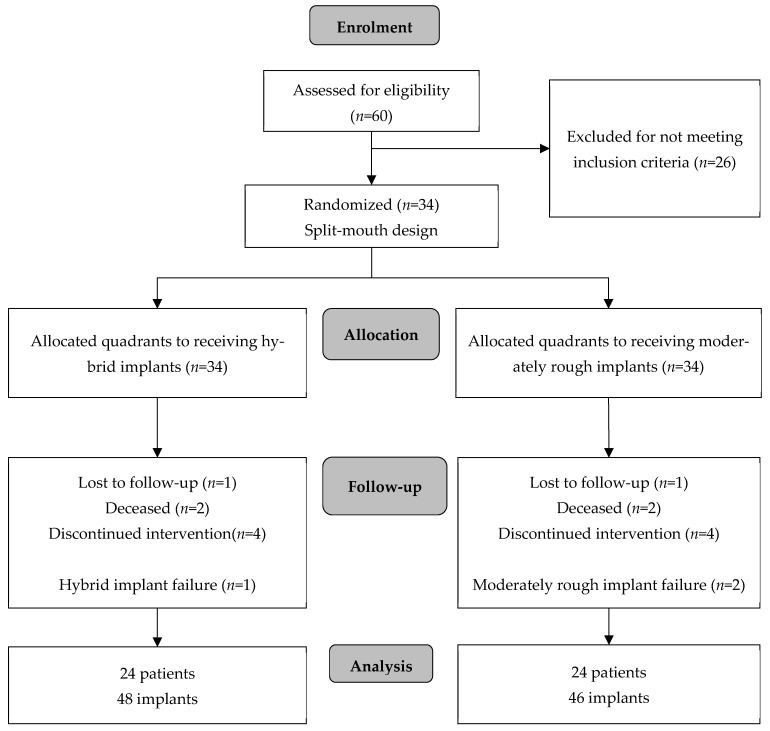
CONSORT flow chart indicating screening visits, randomization, and allocation and numbers or subjects available for data analysis (*n* = number of patients).

The original study cohort consisted of 34 patients requiring treatment according to the inclusion criteria: 11 males and 23 females with a mean age of 66 ± 8.2 years (range = 48–86) participated. Three of the included patients were smokers. At the 4-year recall, 10 patients could not be included in the analysis of the data. Two patients had passed away, 1 had moved countries, and a further 7 patients dropped out by the 4th year, due to unwillingness to be part of the study for medical health reasons. The final group comprised 24 patients: 4 men and 20 women with an average age of 65.1 ± 8.6 years (range= 48–86) (see Figure 2). A total of 94 implants were available for clinical examination: 48 hybrid implants and 46 moderately rough implants.

Average final insertion torque was 56.7 ± 21.1 Ncm (range = 10–80). The implants in 1 patient did not achieve the minimum allowable insertion torque; therefore, the provisional prosthesis was loaded after 6 months. All implants in the remaining patients achieved >25 Ncm, allowing the provisional prostheses to be loaded immediately. The mean follow-up time for measuring clinical indices for T_1_ was 9 ± 4.4 months (range = 3–21 months), and for T_2_ it was 52.6 ± 3.4 months (range = 47–59). See Table S1 in Supplementary Data for the full results.

### 3.1. Crestal Bone Loss

**Figure 3:**
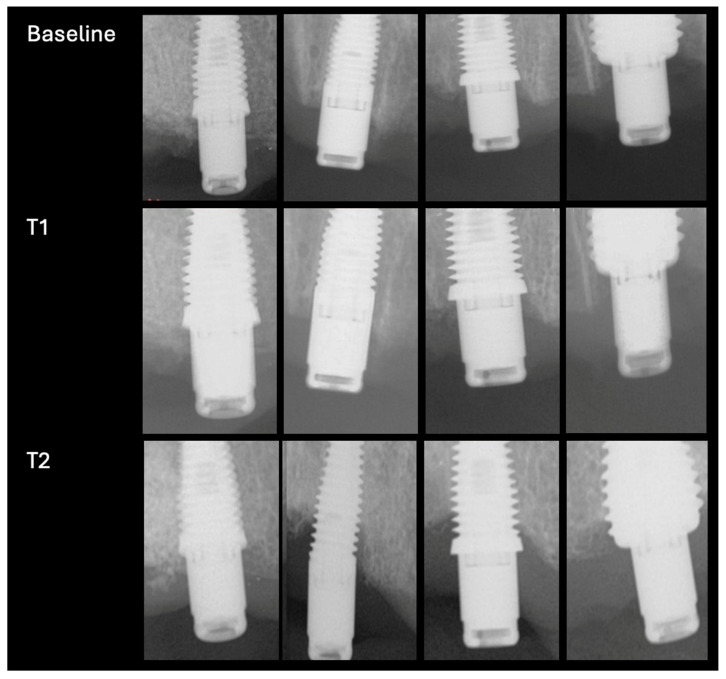
Radiographs of a typical case at baseline, at follow point T1 and at follow up point T2.

The IC coefficient assessing reliability on bone loss measurements was 0.947 (*p* < 0.001), suggesting excellent agreement between the 2 bone level examiners. Radiographs at baseline, at follow-up timepoint T_1_, and at follow-up timepoint T_2_ for a typical case are shown in Figure 3.

**Figure 4:**
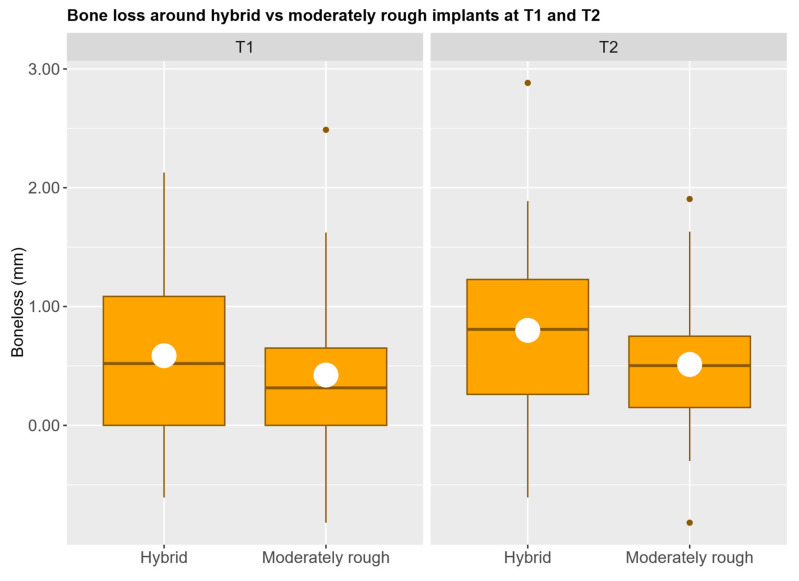
Distribution of bone loss for each implant type at 1 year (T1) and 4 years (T2). Mean bone loss is represented by the white dots.

Mean crestal bone loss was 0.56 ± 0.67 mm in the hybrid group and 0.43 ± 0.61 mm in the moderately rough group at T_1_. Mean crestal bone loss was 0.80 ± 0.65 mm in the hybrid group and 0.59 ± 0.57 mm in the moderately rough group at T_2_. No statistically significant differences were found between the fixed effects in the mixed effects model, see Table 1 and Figure 4.

### 3.2. Implant Survival

Implant survival was 96.9% with three implant failures. One moderately rough implant did not integrate and was removed after 2 months. Two implants from the same patient (one hybrid and one moderately rough) showed considerable buccal gingival recession, deep pocketing, and bleeding on probing, 10 months after placement and were removed. The failed implants were replaced but excluded from further analysis.

### 3.3. Pocket Probing Depth (PPD), Plaque Index (PI), and Bleeding on Probing (BOP)

Mean PPD was 2.38 ± 1.02 mm (range = 0–3.55) in the hybrid implant group and 2.34 ± 1.00 mm (range = 0–3.50) in the moderately rough group. Pockets probing deeper than 5 mm were found in five patients in the hybrid implant group and in three patients in the moderately roughened implant group at T_1_. Mean PPD was 2.03 ± 0.55 mm (range = 1.15–3.40) in the hybrid implant group and 2.17 ± 0.61 mm (range = 1.20–3.50) in the moderately rough implant group at T_2_. At both T_1_ and T_2_, there were no statistically significant differences in PPD between hybrid and moderately rough implants (see Table 2).

Mean PI was 21 ± 22% (range = 0–75) in the hybrid implant group and 20 ± 23% (range = 0–75) in the moderately rough group at T_1_. Mean PI was 9 ± 12% (range = 0–42) in the hybrid implant group and 13% ± 13% (range = 0–42) in the moderately rough group at T_2_. At both T_1_ and T_2_, there were no statistically significant differences in PI between hybrid and moderately rough implants (see Table 2).

Mean BOP was 20 ± 15% (range = 0–50) in the hybrid implant group and 16 ± 18% (range = 0–50) in the moderately rough group at T_1_. Mean BOP was 20 ± 16% (range = 0–75) in the hybrid implant group and 20% ± 19% (range = 0–75) in the moderately rough group at T_4_. At both T_1_ and T_2_, there were no statistically significant differences in BOP between hybrid and moderately rough implants (see Table 2).

## 4. Discussion

In the present study, limited crestal bone loss (hybrid group: 0.80 ± 0.65 mm; moderately rough group: 0.59 ± 0.57 mm) and a high implant survival rate (96.9%) were found after 52.6 months. No statistically significant difference in crestal bone loss was found between the moderately rough and hybrid surface implants, irrespective of the timing of implant placement, final prosthetic rehabilitation, or implant angulation. The prosthetic protocol consisted of immediate loading with a removable denture. Considering that 63% of the implants were immediately placed, there was an increased risk of early implant failure due to overloading during the bone healing and implant integration phase. By placing locator abutments and attaching the prosthesis immediately after implant placement, a kind of implant splinting was achieved. The temporary restoration was then relined over these healing caps, allowing direct contact between the implants and the prosthesis. A similar implant survival of 96% was reported, suggesting that immediate loading of 4 implants with a removable prosthesis could be considered as a viable treatment alternative, avoiding a second stage surgery and leading to a faster function for the patient.

Studies evaluating crestal bone loss and peri-implant health parameters at hybrid surface implants with a follow up of at least 3 years are scarce. Tirone and colleagues performed a split-mouth study in which patients received two single implants (a machined surface implant and a moderately rough surface implant) [27]. Implants from two different manufacturers were used in this study; however, each patient received implants from the same manufacturer. After 3 years of follow-up, no significant difference in crestal bone loss was observed between the implants with different surfaces (*p* = 0.12). However, Tirone and colleagues found that bone loss differed significantly between the implants from the different manufacturers (*p* = 0.007). This result suggests that additional factors, such as implant neck design, have a greater influence on crestal bone loss than the implant surface alone. In another split-mouth study conducted by Raes and colleagues, patients previously diagnosed with severe periodontitis received at least one minimally rough implant and one moderately rough implant [20]. At the 5-year follow up appointment, the minimally rough implants showed slightly less crestal bone loss compared to the moderately rough implants (1.0 vs. 1.7, *p* = 0.06), suggesting that a smoother surface may have a benefit in patients with a higher peri-implantitis risk. Conversely, Lee and colleagues found significantly greater crestal bone loss at hybrid surface implants after 3 years (*p* < 0.001) [28]. However, when examining the implants used in the study more closely, the moderately rough implant was a tapered implant featuring micro threads at the coronal aspect, while the hybrid implant was a straight implant without an external thread at the coronal aspect. This difference in macro-design may be as important, if not more, than the implant surface. In another long-term study comparing hybrid implants with moderately rough implants by Zetterqvist et al., a statistically significant increase in bone loss was found at the hybrid implants (*p* < 0.0001), which is contradictory to the results of the present study [29]. A possible explanation for this difference is that the machined section of the hybrid implants from Zetterqvist had an *S_a_* value of 0.18 µm, which is very smooth when compared to the machined section of the hybrid implants used in the present investigation (*S_a_* = 0.9 µm). This difference in roughness may also suggest that a certain roughness is required to preserve the crestal bone.

A major limitation of the current study is the lack of statistical power due to the high dropout rate. This can mainly be attributed to the global COVID-19 pandemic, as 7 of the original patients declined to participate in the study for medical reasons. The heterogeneous nature of the study population (placing implants in healed and extraction sites; using implants with and without angulation correction; including grafted and non-grafted implant sites; using fixed versus removable prostheses) may also be considered as a limitation when comparing crestal bone level changes around hybrid and moderately rough implants. While the linear mixed effects model attempted to account for these confounding factors, the limited crestal bone loss and high implant survival results show that hybrid implants are still a predictable treatment option.

## 5. Conclusions

Considering the sample size-related limitations of this randomized controlled trial, both hybrid and moderately rough implants showed minimal bone loss over a 4-year follow up period. While these results would suggest comparable stability over the longer term, the limited sample size prevents definitive conclusions from being made regarding clinical superiority and broader generalizability of the results. A larger, adequately powered study is warranted to confirm these findings.

## Figures and Tables

**Table 1:** Results from the linear mixed effects model analysis estimating the effect of implant type, implant angulation, site type and grafting on crestal bone loss at T1 and T2.

Time Point	Parameter	Implant Group	Estimated Marginal Means	Standard Error	95% Confidence Level		*p*-Value
					Upper Bound	Lower Bound	
T_1_	Implant type	Hybrid	0.57	0.17	0.24	0.91	0.716
		Moderately rough	0.54	0.16	0.22	0.85	
	Implant angulation	Hybrid	0.53	0.17	0.18	0.87	0.685
		Moderately rough	0.16	0.16	0.26	0.90	
	Surgery type	Hybrid	0.55	0.16	0.24	0.87	0.999
		Moderately rough	0.55	0.18	0.19	0.92	
	Graft	Hybrid	0.50	0.25	0.00	1.00	0.644
		Moderately rough	0.61	0.11	0.38	0.84	
	Restoration type	Hybrid	0.51	0.20	0.11	0.92	0.710
		Moderately rough	0.60	0.17	0.25	0.95	
T_2_	Implant type	Hybrid	0.77	0.14	0.49	1.06	0.074
		Moderately rough	0.57	0.14	0.30	0.84	
	Implant angulation	Hybrid	0.68	0.15	0.37	0.98	0.892
		Moderately rough	0.66	0.14	0.39	0.93	
	Surgery type	Hybrid	0.75	0.15	0.44	1.05	0.282
		Moderately rough	0.60	0.14	0.32	0.87	
	Graft	Hybrid	0.53	0.23	0.07	0.98	0.217
		Moderately rough	0.81	0.08	0.64	0.99	
	Restoration type	Hybrid	0.67	0.14	0.40	0.95	0.979
		Moderately rough	0.67	0.17	0.34	1.00	

**Table 2:** Summary of the pocket probing depth (PPD), plaque index (PI) and bleeding on probing (BOP) results.

Parameter	Implant Group	T_1_				T_2_			
		Mean	SD	Range	*p*-Value	Mean	SD	Range	*p*-Value
PPD (mm)	Hybrid	2.38	1.02	0–3.55	0.564	2.04	0.57	1.15–3.40	0.295
	Moderately rough	2.34	1.00	0–3.50		2.18	0.61	1.20–3.50	
PI (%)	Hybrid	22	23	0–83	0.830	9	13	0–42	0.076
	Moderately rough	22	23	0–75		13	13	0–42	
BOP (%)	Hybrid	20	19	0–75	0.214	9	12	0–42	0.637
	Moderately rough	16	18	0–67		20	19	0–75	

## Data Availability

The original contributions presented in this study are included in the article/Supplementary Materials. Further inquiries can be directed to the corresponding author.

## Supplementary Materials

The following supporting information can be downloaded at: https://www.mdpi.com/article/10.3390/jcm14227996/s1, Table S1: SupportingData; Table S2: CONSORT_checklist.

## Abbreviations

The following abbreviations are used in this manuscript:

*Sa*	Arithmetical mean height of surface area
*S_dr_*	Developed interfacial area ratio
mm	Millimeters
Ncm	Newton centimeters
PPD	Pocket Probing Depth
PI	Plaque Index
BOP	Bleeding on Probing
